# A spatio-temporal dataset for ecophysiological monitoring of urban trees

**DOI:** 10.1016/j.dib.2024.111010

**Published:** 2024-10-09

**Authors:** Théo Le Saint, Jean Nabucet, Cécile Sulmon, Julien Pellen, Karine Adeline, Laurence Hubert-Moy

**Affiliations:** a Université Rennes 2, LETG Rennes UMR CNRS 6554 LETG, Place du recteur Henri Le Moal, 35043 Rennes cedex, France; b Univ Rennes, CNRS, ECOBIO [(Ecosystèmes, biodiversité, évolution)] UMR 6553, Rennes, France; c ONERA-DOTA, University of Toulouse, FR-31055 Toulouse, France

**Keywords:** Tree rows, Foliar ecophysiological traits, Tree plant area density, Phenological monitoring

## Abstract

A dataset was produced for 117 urban trees in four monospecific tree rows in the city of Rennes, northwestern France. The trees were measured in nine 2- to 3-day measurement sessions from Apr-Sep 2021. The dataset includes (i) leaf traits (*i.e.*, contents of pigments, water and dry matter) measured *in situ* and in the laboratory; (ii) plant area density measured *in situ* under the canopy and (iii) georeferenced data that describe the location, geometry and species of the trees. The dataset provides an original overview of dynamics of the contents of pigments, water and dry matter and plant area density for four tree species grown under urban conditions. It can be used for several purposes, such as identifying trees’ responses/behaviors in relation to their urban environment or climate conditions.

Specifications Table

**Table:** 

Subject	Plant Science: Plant Physiology
Specific subject area	Ecophysiological monitoring
Type of data	Raw, Analyzed, Georeferenced point data
Data collection	The dataset describes 117 urban trees of four species (*Platanus acerifolia, Acer platanoides, Fraxinus excelsior* and *Quercus rubra*). The trees were measured in nine 2- to 3-day sessions during the growing season (Apr-Sep) in 2021. Three types of data were collected: (i) Leaf traits (*i.e.*, contents of pigments, water content and dry matter) were measured on leaves *in situ* and in laboratory. Several methods were used to measure pigment contents: chlorophylls (C_ab_), flavonols (Flav), anthocyanins (Anth) and the nitrogen balance index (NBI) were measured *in situ* using an optical leafclip meter (Dualex 4 Scientific, Force-A, Orsay, France). chlorophyll *a* (C_a_)*,* chlorophyll *b* (C_b_)*,* chlorophyll *a+b* (C_ab_) and carotenoid (Car) contents were determined in sampled leaves according to Lichtenthaler [1], and anthocyanin content was determined according to Gravot [2]; To determine leaf contents of water and dry matter, fresh leaves were weighed *in situ* to measure fresh weight and then dried and reweighed to measure dry weight. (ii) Plant area density (PAD) measurements were taken at the tree scale using a plant canopy analyzer (LAI-2200, LI-COR, Lincoln, NE, USA) using four methods included in FV-2200 software (v.1.2, LI-COR). (iii) Georeferenced points for the trees studied were obtained from the OpenData database of Rennes Métropole.
Data source location	City /Region: Rennes, Brittany Country: France Latitude and longitude: *48.11°N, 1.68°W*
Data accessibility	Repository name: Zenodo Data identification number: 10.5281/zenodo.12751353 Direct URL to data: https://doi.org/10.5281/zenodo.12751353 The primary data for the canopy-height model (CHM) at 0.5 m spatial resolution are available in raster format from Rennes Métropole as follows: Repository name: OpenData Rennes Métropole Data identification number: mnt_2021_arbres Direct URL to data: https://data.rennesmetropole.fr/explore/dataset/mnt_2021_arbres/information/ The primary data for the digital terrain model (DTM) at 0.5 m spatial resolution are available in raster format from Rennes Métropole as follows: Repository name: OpenData Rennes Métropole Data identification number: mnt_2021_sol Direct URL to data: https://data.rennesmetropole.fr/explore/dataset/mnt_2021_sol/information/ The primary data for orthophotography at 0.05 m spatial resolution are available in raster format from Rennes Métropole as follows: Repository name: OpenData Rennes Métropole Data identification number: orthophotographie-2021 Direct URL to data: https://data.rennesmetropole.fr/explore/dataset/orthophotographie-2021/information/ The primary data for the locations of tree rows are available in vector format from Rennes Métropole as follows: Repository name: OpenData Rennes Métropole Data identification number: arbres-d-alignement-rennes Direct URL to data: https://data.rennesmetropole.fr/explore/dataset/ arbres-d-alignement-rennes/information/
Related research article	*–*

## 1 Value of the Data

• The data provide an overview of dynamics of the contents of pigments, water and dry matter for four tree species grown under various urban conditions over the entire growing season of 2021.
• The data can be used to identify trees’ responses/behaviors in relation to their urban environment (the imperviousness and building density, the surrounding urban materials,..) and climate conditions (temperatures, precipitations).
• The georeferenced data were collected for use in remote sensing applications. They can be used as training or test samples for modeling functional traits of trees in urban environments. Specifically, they can be used as validation data for vegetation trait estimation or to study relationships between spectral values and vegetation traits in urban environments. The size of the crowns of the selected trees (at least 10 m in diameter) is suited to the use of satellite data such as Sentinel-2 (10-meters spatial resolution) or PlanetScope (3-meters spatial resolution) imagery, and allows vegetation traits to be related to the spectral response of the trees.
• The data can be used for species-specific calibration of Dualex 4 Scientific measurements. Specifically, chlorophyll and anthocyanin were measured both *in situ* (Dualex) and in the laboratory. For the four deciduous species examined in his study, no Dualex calibration equation is available to date.
• The georeferenced data can be used as predictor variables for spatial modeling of urban tree health.
• The data can benefit scientists and stakeholders in the fields of ecology, remote sensing or urban planning.

## 2 Background

The dataset presented in this study was created to monitor trees in urban environments and to be used for remote sensing applications, in particular to validate vegetation products (leaf/plant area index, density and chlorophyll content estimation) derived from Sentinel-2 imagery. The data collection protocol was designed to be replicable in other cities, using data, methods and equipment that are easy to use and readily available. *In particular, we used a DTM and a CHM for modelling the geometry of the tree crown, which are available in many cities*. Sampling was carried out to cover several species, with a significant intra-annual temporal dimension to track the dynamics of the observed vegetation traits.

## 3 Data Description

In total, 117 trees of four species (*Acer platanoides* (AC)*, Fraxinus excelsior* (FR)*, Platanus acerifolia* (PL) *and Quercus rubra* (QR)) were measured in the city of Rennes (Brittany, France) in nine 2- to 3-day sessions during the growing season (Apr-Sep) in 2021. Part 1 of the dataset contains measurements of leaf traits (*i.e.*, contents of pigments, water and dry matter) (Table 1). Two methods were used to determine leaf pigment contents:

• Leaves were sampled *in situ*, and pigment contents (*i.e.*, chlorophylls, anthocyanins and flavonols) and the nitrogen balance index (NBI) were measured non-destructively using an optical leafclip meter (Dualex 4 Scientific, Force-A, Orsay, France, https://metos.global/en/dualex/). Accuracy of Dualex measurement was assessed by Cerovic et al., 2012. Results showed a very high reproducibility with a RMSE of 0.713 **µ**g/cm² (comparison based on measurement carried on the same leaf with five Dualex 4 devices) and a very high overall accuracy with a RMSE of 5.03 µg/cm² (comparison with chlorophyll extraction in laboratory) [3].
• Leaves sampled *in situ* were stored at −20 °C, freeze-dried, and then pigments were extracted in the laboratory using the Lichtenthaler [1] method for chlorophylls and carotenes, and the Gravot [2] method for anthocyanins.

**Table 1:** Description of the dataset (part 1). LM: Lichtenthaler method.

Variable	Description	Unit/Format
TREE_ID	Unique tree ID	–
DATE	Measurement date	YYYY-MM-DD
SP	Tree species	–
C_a__LAB	Chlorophyll *a* content calculated using the LM	µg/mg DW
C_b__LAB	Chlorophyll *b* content calculated using the LM	µg/mg DW
C_ab__LAB	Total chlorophyll (*a* + *b*) content (*i.e.*, Ca_LAB + Cb_LAB)	µg/mg DW
Car_LAB	Carotenoid content calculated using the LM	µg/mg DW
ANTH_LAB	Anthocyanin content calculated using the Gravot method	DO_532_/ml/mg DW
Cab_DX	Chlorophyll content measured using the Dualex 4 Scientific	µg/cm²
ANTH_DX	Anthocyanin content measured using the Dualex 4 Scientific	Arbitrary unit
FLV_DX	Flavonol content measured using the Dualex 4 Scientific	Arbitrary unit
NBI_DX	Nitrogen balance index measured using the Dualex 4 Scientific	Arbitrary unit
LDMC	Leaf dry matter content	%
LWC	Leaf water content	%
FW	Fresh weight	g
DW	Dry weight	g

**Fig. 1:**
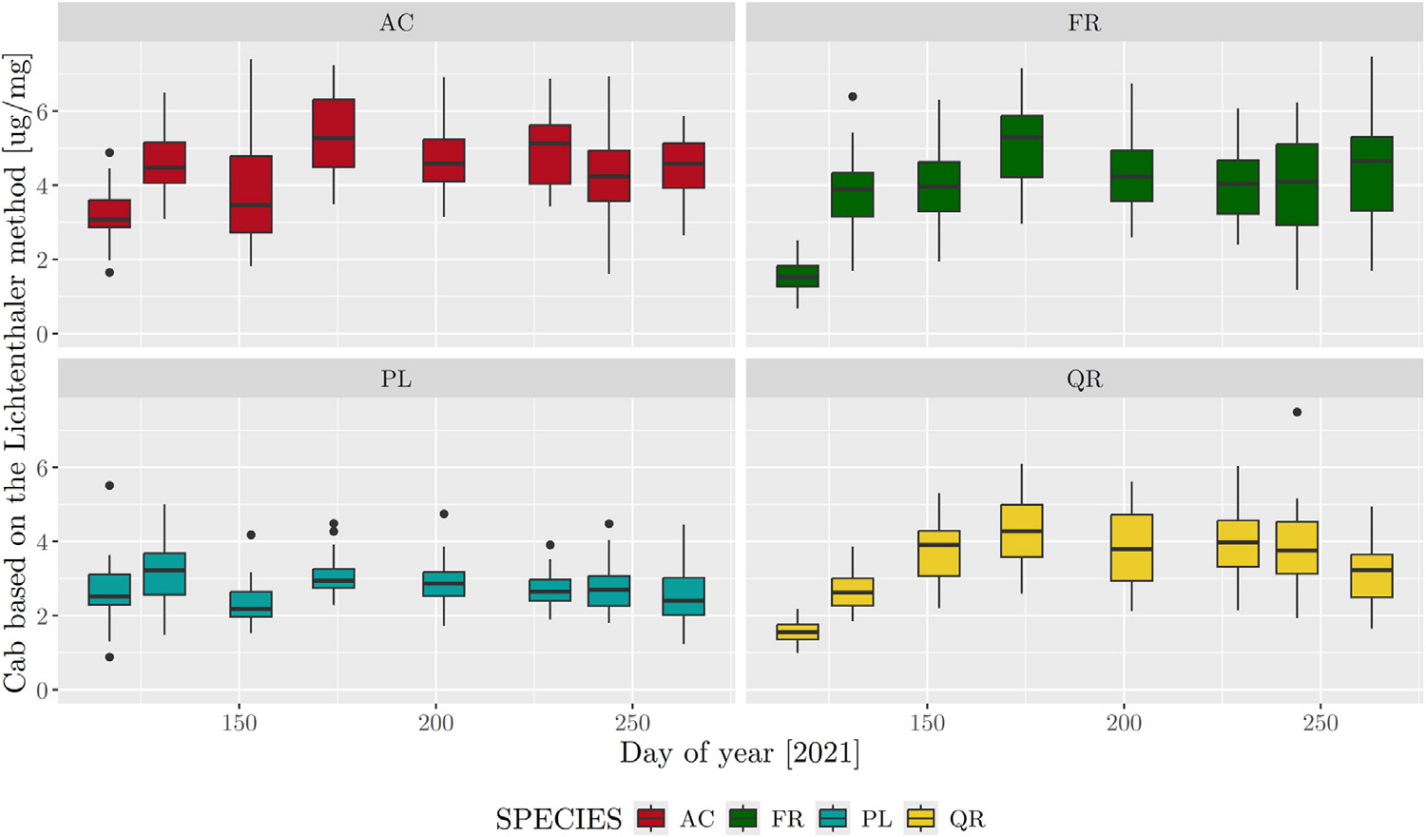
Boxplots of total chlorophyll (*a* + *b*) content (Cab) calculated using the Lichtenthaler method during the growing season for the four tree species (AC: *Acer platanoides*; FR: *Fraxinus excelsior*; PL: *Platanus acerifolia*; QR: *Quercus rubra)*. Whiskers equal 1.5 times the interquartile range.

Considering the entire sampling period (13 Apr to 20 Sep 2021), total chlorophyll (*a* + *b*) content calculated using the Lichtenthaler method ranged from 0.7 to 10.4 **µ**g/g dry weight (DW), with a mean and median of 3.6 and 3.4 µg/mg DW, respectively. Species-specific means were 2.6, 3.4, 4.0 and 4.4 **µ**g/g DW for PL, QR, FR and AC, respectively. AC, FR and QR had higher variability during the growing season (medians of 1.56–5.30 µg/g DW) than PL did (medians of 2.28–3.23 **µ**g/g DW) (Fig. 1).

**Fig. 2:**
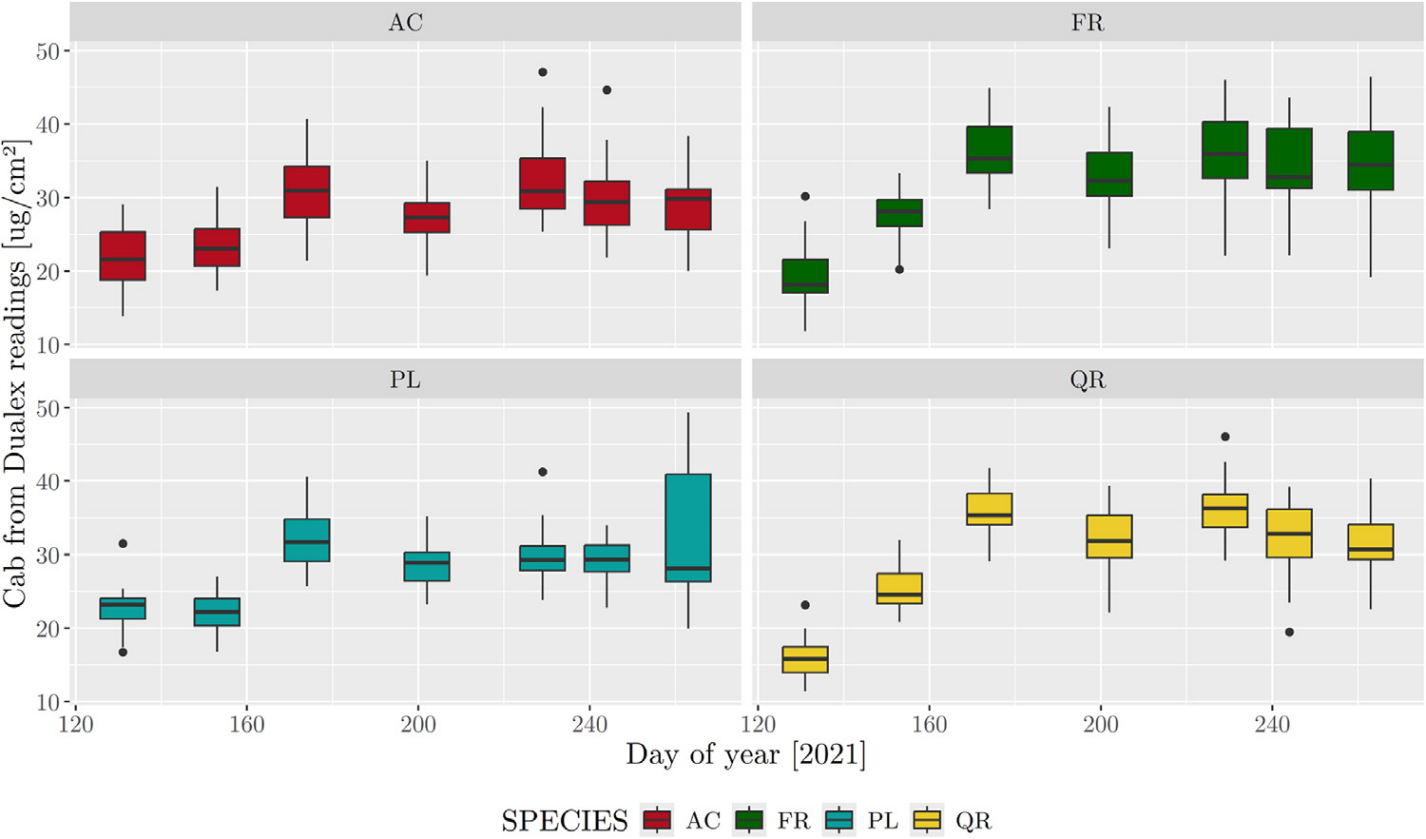
Boxplots of chlorophyll content (Cab) measured using the Dualex 4 Scientific during the growing season for the four tree species (AC: *Acer platanoides*; FR: *Fraxinus excelsior*; PL: *Platanus acerifolia*; QR: *Quercus rubra*). Whiskers equal 1.5 times the interquartile range.

Chlorophyll content measured using the Dualex 4 Scientific (measured from 11 May to 20 Sep 2021) ranged from 11.4 to 49.3 **µ**g/cm², with a mean and median of 29.4 for both (Fig. 2). Species-specific means were 27.5, 27.9, 31.1 and 32.0 **µ**g/cm² for AC, PL, QR and FR, respectively. FR and QR increased to a median content of *ca*. 35 **µ**g/cm² on day of year 153 (2 Jun), which contrasted with the relatively smaller increase to 31 **µ**g/cm² observed for PL and AC.

Part 2 of the dataset contains the plant area density (PAD) measured using a plant canopy analyzer (LAI-2200, LI-COR, Lincoln, NE, USA) (Table 2). For each tree and date, four measurements were taken under the canopy (one in each cardinal direction). PAD was calculated at the tree-crown scale using four methods included in FV-2200 software (v.1.0.0, LI-COR): the “LAI-2200 method”, which is the default method used in FV-2200 (described in the user manual https://edisciplinas.usp.br/pluginfile.php/4857358/mod_resource/content/0/Manual-LAI-2200-EN.pdf), the Lang method [4], the ellipsoidal method [5] and the constrained least-squares method [6].

**Table 2:** Description of the dataset (part 2). PAD: plant area density.

Variable	Description	Unit/Format
TREE_ID	Unique tree ID	–
FACE	Face of the tree measured	*N*=north; *E*=east; *S* = south; *W*=west
DATE	Measurement date	YYYY-MM-DD
SP	Tree species	–
PAD	PAD calculated using the LAI-2200 method	m^2^/m^3^
LangPAD	PAD calculated using the Lang method	m^2^/m^3^
EllipPAD	PAD calculated using the ellipsoidal method	m^2^/m^3^
ClsPAD	PAD calculated using the constrained-least squares method	m^2^/m^3^

**Fig. 3:**
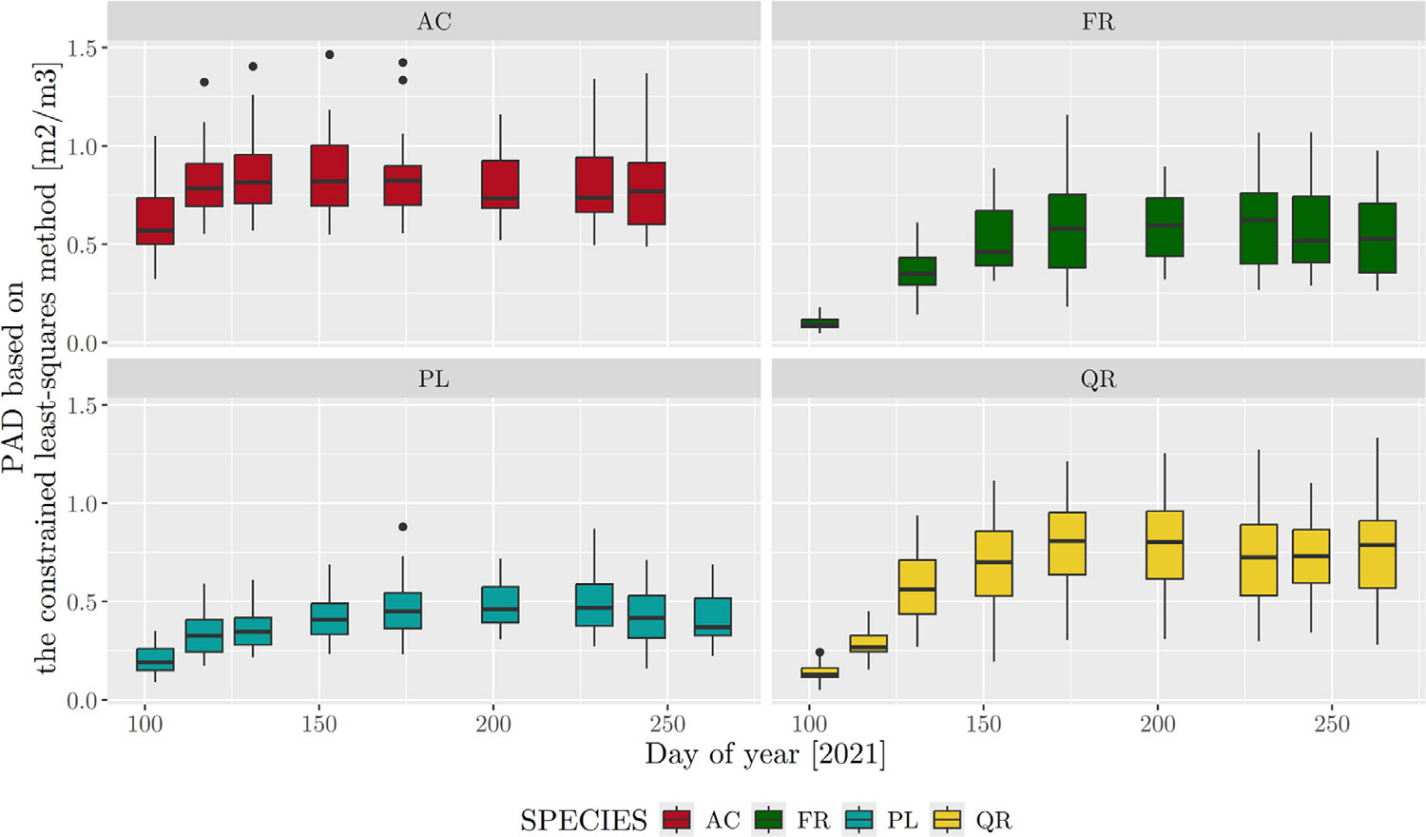
Boxplots of plant area density (PAD) calculated using the constrained-least squares method during the growing season for the four tree species (AC: *Acer platanoides*; FR: *Fraxinus excelsior*; PL: *Platanus acerifolia*; QR: *Quercus rubra*). Whiskers equal 1.5 times the interquartile range. Because the PAD data may contain outliers due to sensor malfunctions, the boxplots exclude values greater than the 97th percentile. The PAD value for a given tree corresponds to the mean of the tree faces’ PAD values.

PAD calculated using the constrained-least squares method ranged from 0.04 to 1.46 **m²**/m^3^, with a mean and median of 0.57 and 0.56 **m²**/m^3^, respectively (Fig. 3). Species-specific means were 0.38, 0.44, 0.67 and 0.76 for PL, FR, QR and AC, respectively. LAD for FR and QR had a similar trend and amplitude. LAD for AC and PL also had a similar trend, although AC had higher medians (0.57 −0.82 **m²**/m^3^) than PL did (0.19–0.47 **m²**/m^3^).

Part 3 of the dataset is a geopackage (gpkg) data file (Projection WGS 84/UTM zone 30 N) that contains two spatial layers that describe the location, geometry and species of the trees (Table 3). The point layer (TRUNK_LOCATION) and the polygon layer (CROWN_EXTENT) represent the trunk location and crown extent of the tree, respectively. The former was extracted from a dataset of tree-row locations, while the latter was manually digitized from a canopy-height model and 2021 orthophotography.

**Table 3:** Description of the dataset (part 3).

Variable	Description
TREE_ID	Unique tree ID
GENUS	Tree genus
SPECIES	Tree species (specific epithet)
SITE	Site abbreviation

Several types of data were collected (Table 4). Depending upon the equipment available and weather conditions, some measurements were not taken for certain sites/dates.

**Table 4:** Description of the variables measured (green “V” cells) or not measured (red “X" cells) (*sources in italics*) for the four tree species (AC: *Acer platanoides*; FR: *Fraxinus excelsior*; PL: *Platanus acerifolia*; QR: *Quercus rubra*). NBI: Nitrogen balance index.

## 4 Experimental Design, Materials and Methods

### 4.1 Study sites

**Fig. 4:**
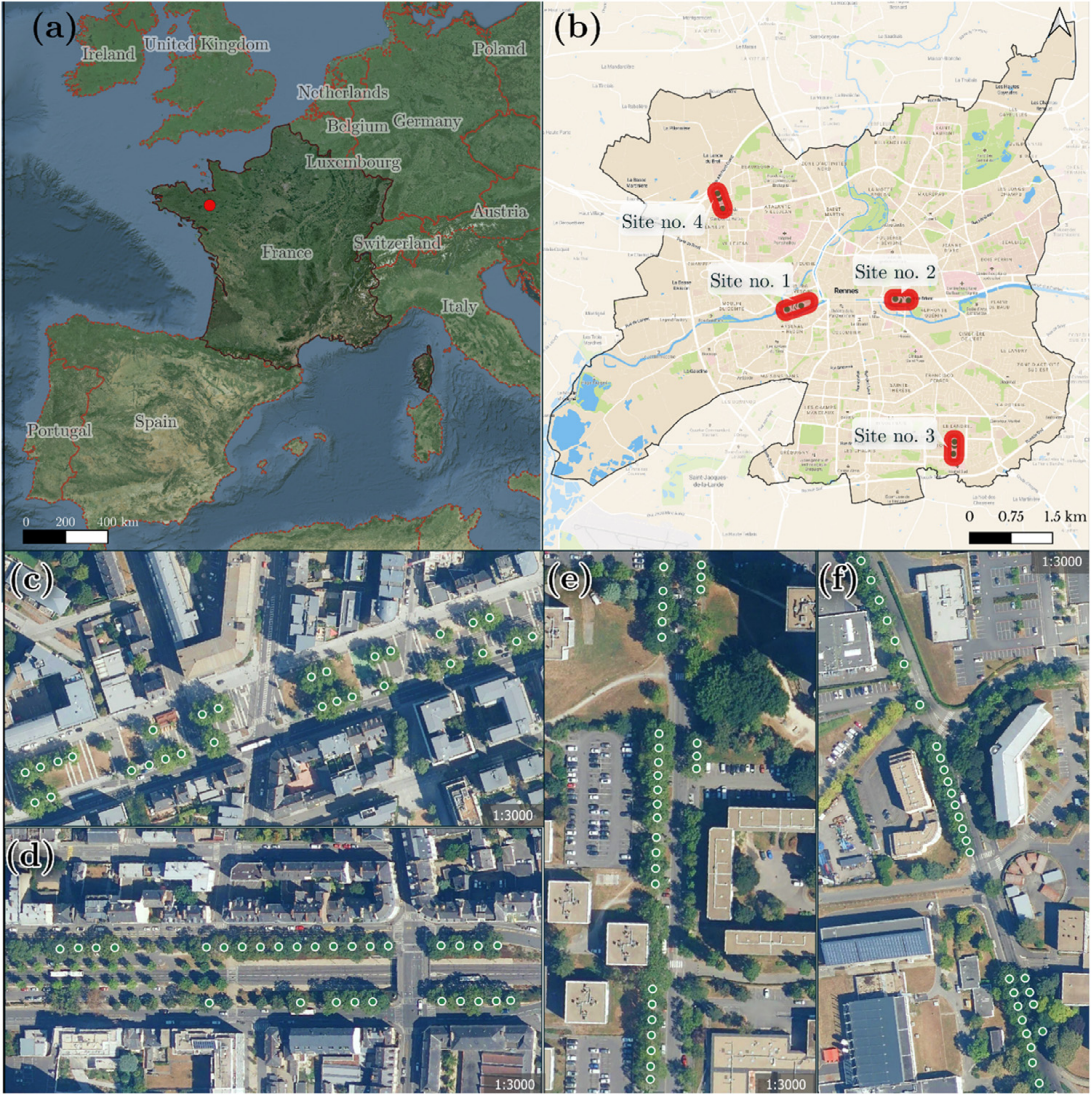
Map of the four study sites. Locations of (a) the city of Rennes in western Europe, (b) the four study sites (tree rows) in Rennes and tree rows no. (c) 1, (d) 2, (e) 3 and (f) 4.

In total, 117 trees at four sites of monospecific tree rows were measured in the city of Rennes, northwestern France (Table 5, Fig. 4).

**Table 5:** Description of the four sites of urban tree rows.

Tree row	Site name	Site abbreviation	No. of trees	Species scientific name	Species common name
1	Mail François Mitterrand	MAIL	29	*Platanus acerifolia*	London planetree
2	Avenue du Sergent Maginot / Aristide Bruant	MAGINOT	29	*Acer platanoides*	Norway maple
3	Rue de Roumanie	ROUMANIE	29	*Fraxinus excelsior*	European ash
4	Rue Doyen Denis Leroy	CNRS	30	*Quercus rubra*	Northern red oak

Rennes, which is located in the North-West of France (48.1°, −1.68°) (Fig. 4a), is a medium-sized city of 222,485 inhabitants, with a population density of 4414 people/km2 (Institut national de la statistique et des études économiques, 2020 census). The city covers approximately 50.39 km^2^ and includes distinct areas with various urban densities and vegetation cover types. Almost 130,000 trees were identified by urban tree managers in public spaces in 2021. Most of these trees were planted in rows along the streets.

The four sites were selected in collaboration with urban tree managers from the Direction des Jardins et de la Biodiversité). The four species selected for this study were among the five most common species in Rennes. Due to their strong presence, these species present a major challenge to managers in the context of climate change and urban greening. One tree row was selected for each species, proximity between trees ensuring that they have grown in similar local edaphic and micro-climatic conditions. Only trees with a minimum crown diameter of 10 m (from the zenith) were selected in order to be consistent with remote sensing data, in particular Sentinel-2 images, which have a spatial resolution of 10 m. The health status of the trees was assessed by urban tree managers through a health status score based on visual tree assessment (VTA) [6] and quantified tree risk assessment (QTRA) [7] methods. This score can be found in the *arbres-d-alignement-rennes* primary dataset (diag_phyto). The sites presented heterogeneous levels of health status, ranging from “Trees with minor symptoms that may evolve and present risks” to “Trees that are healthy and growing well”.

**Fig. 5:**
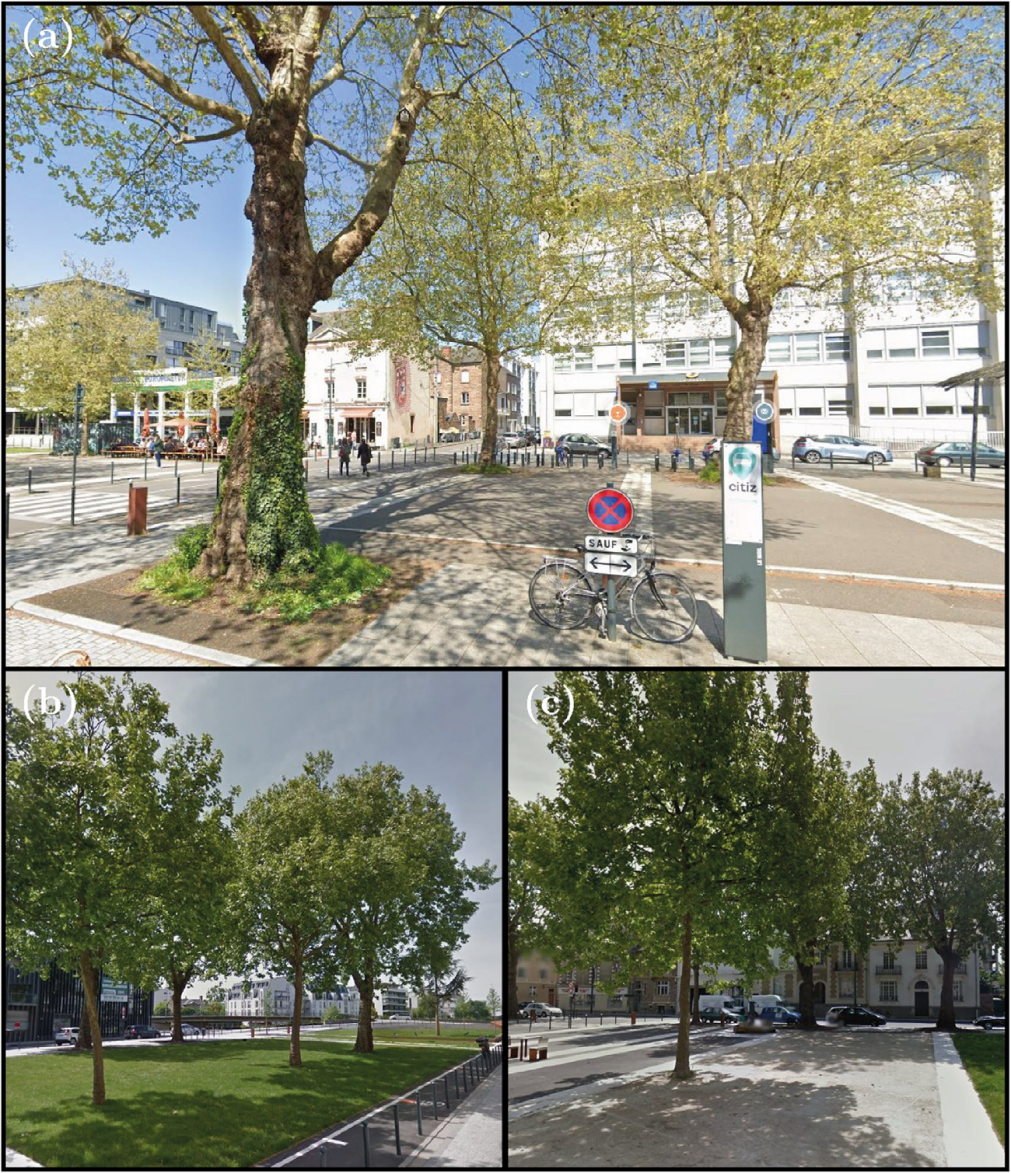
Photos of trees at Mail François Mitterrand (Site no. 1). (a) Tree surrounded by granite and asphalt: foreground tree PL23, background from left to right tree PL06 and tree PL07. (b) Tree planted on lawn: in foreground, from left to right, PL03, PL02 and PL01 trees. In the background, from left to right, PL28 and PL29 trees. (c) Tree planted on stabilized ground: in the foreground tree PL05, in the background from left to right PL24 and PL25 trees. Photo credit: Google Street view.

The first site is a wide street, that corresponds to an urban canyon 700 m long and 50 m wide, with average building heights ranging from 10 to 18 m. The street is bordered by a road to the south and a pedestrian and cycling walkway to the north, and the central section is pedestrian with lawn and asphalt cover. The 29 studied trees are planted on bare ground covered with metal mesh and surrounded by asphalt or granite (Fig. 5a), lawn (Fig. 5b) or stabilized soil (Fig. 5c).

**Fig. 6:**
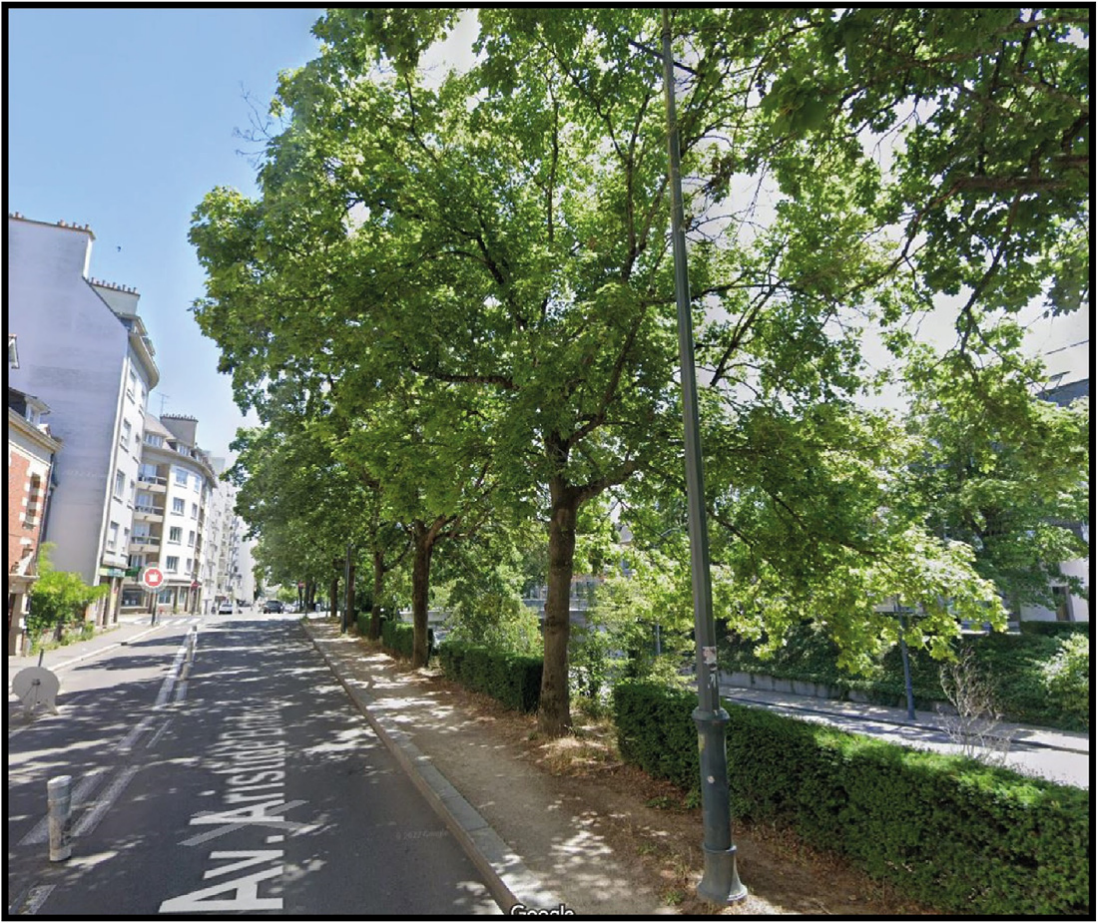
Photo of trees at Avenue Aristide Bruant (Site no. 2). From right to left, along the perspective to the crossroads: AC21, AC22, AC23, AC24, AC25 trees. Photo credit: Google Street view.

The second site is also an urban canyon, 450 m long and 55 m wide. The site is composed of three parallel roads, the central one being bordered by embankments with trees (Fig. 4d). The trees are planted on a stabilized surface with a narrow grass strip and bush hedges (1 meter high) between them (Fig. 6).

**Fig. 7:**
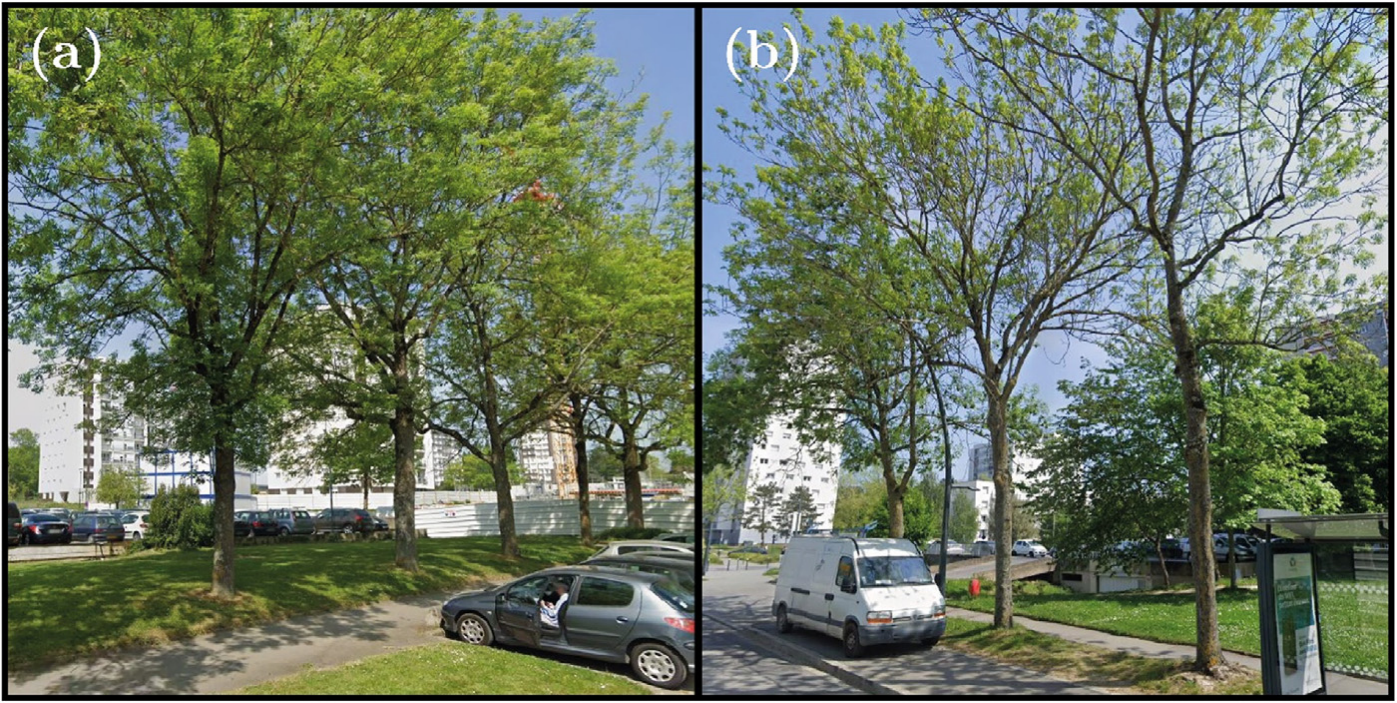
Photos of trees at Rue de Roumanie (Site no. 3). (a) Trees planted on a lawn, from left to right: FR10, FR09, FR08, FR07 and FR06 trees. (b) Trees planted on a grass strip, from right to left: FR27, FR28 and FR29 trees. Photo credit: Google Street view.

The third site corresponds to a more open area with a lower density of buildings and a higher density of vegetated areas. The street is 330 m long and bordered by buildings or lawns. All trees are planted either directly on a lawn (Fig. 7a) or on a grassy strip (Fig. 7b).

**Fig. 8:**
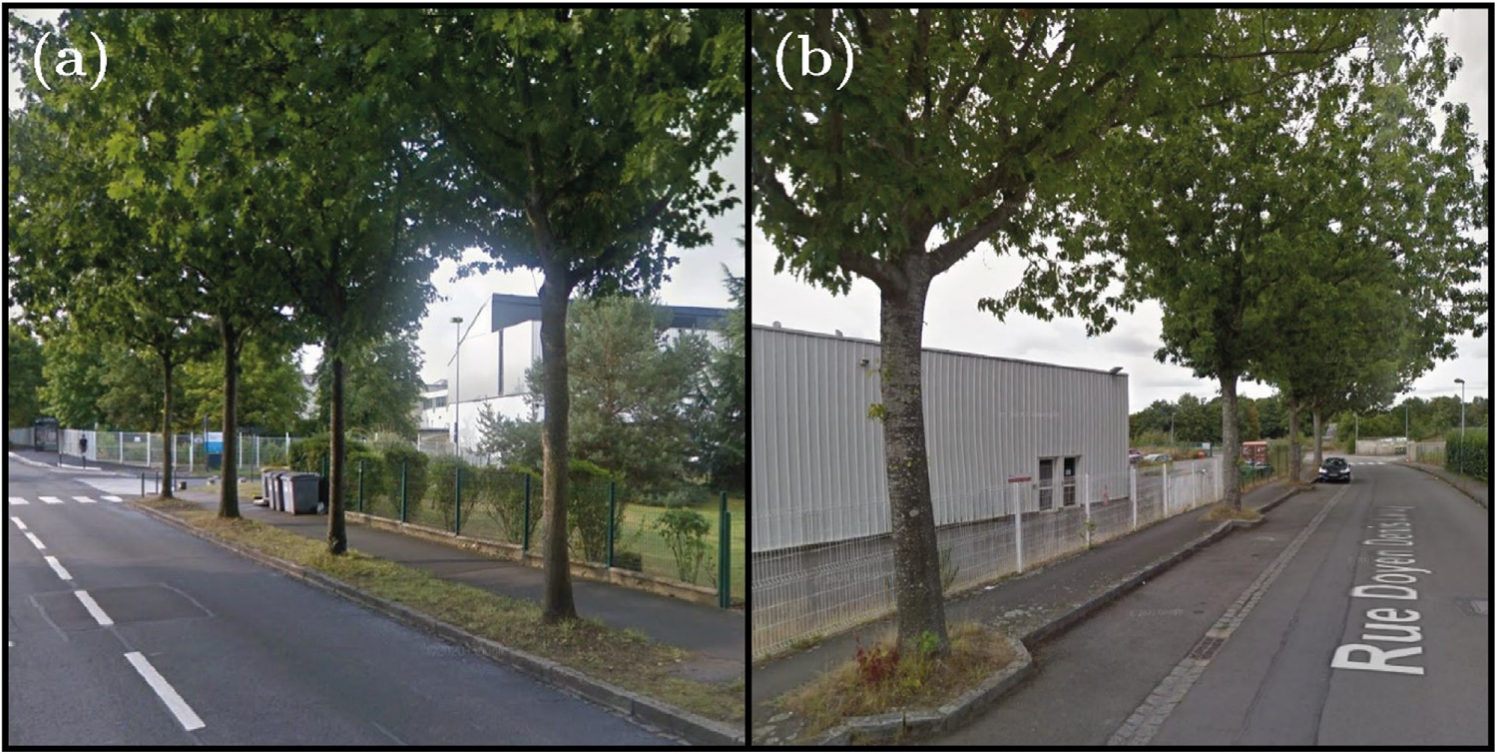
Photos of trees at Rue Doyen Denis Leroy (Site no. 4). (a) Trees planted on a grass strip, from left to right: QR17, QR16, QR15 and QR14 trees. (b) Trees planted on grass plots, from left to right: QR04, QR03, QR02 and QR01 trees. Photo credit: Google Street view.

The fourth site corresponds to two tree rows, with a street of 330 m long. Located in a more industrial area than the other sites, the density of residential housing is lower including several service or industrial buildings. The trees are planted on a grass strip (southern part, Fig. 8a) or on grass plots integrated into the sidewalk (northern part, Fig. 8b). The site is located 150 m from the Rennes ring road.

### 4.2 Measurement sessions and climatic conditions

**Fig. 9:**
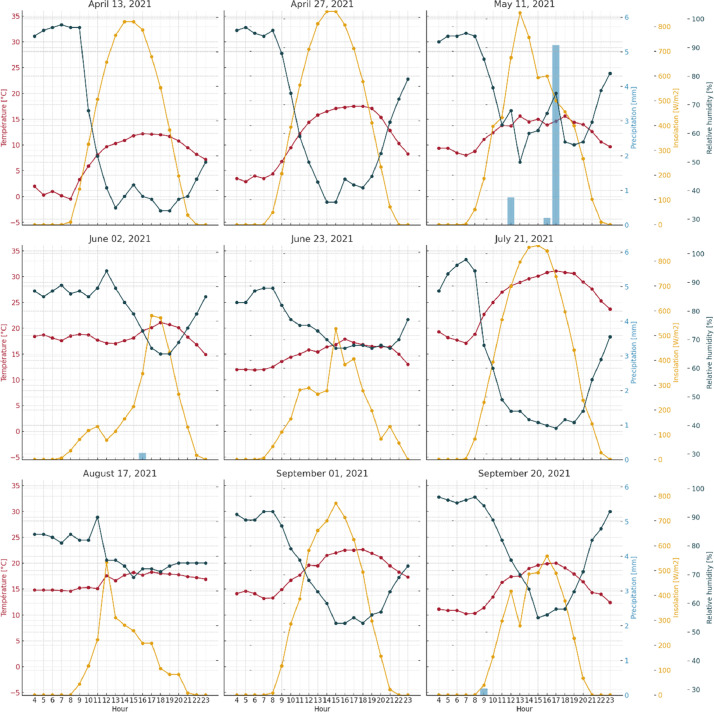
Hourly climatic time series (between 4 a.m. and 11 p.m.) for each *in situ* measurement date. Temperature curves are shown in red, insulation in orange and relative humidity in dark blue. Precipitation was represented with light blue bars. Climatic data were obtained from Météo France's 'Rennes-St Jacques' weather station, located at Saint-Jacques de la Lande, south-west of Rennes, 48.07°N; 1.74°W.

A typical one-day measurement session was organized as follows: (i) PAD was measured in the early morning (between 5am and 9am) at site n°4 then site no. 1; (ii) leaf collection and *in situ* measurements were carried out at site no. 1 from 9am to 1pm and at sites no. 2, 3 and 4 from 2pm to 7:30pm; (iii) Evening PAD measurement was conducted at sites no. 2 and 3. Due to variations in sunrise and sunset times over the measurement sessions, PAD measurements at site no. 4 were sometimes postponed to the following morning, and those at site no. 2 to the following evening. The measurement dates were chosen to cover the entire growing period, from the green-up phase and the appearance of the first leaves to the beginning of senescence phase. The measurement dates were distributed across these two phenological phases in order to obtain uniform temporal coverage of the growing season. The exact dates were determined according to three criteria: matching with Sentinel-2 image acquisition dates, free-cloud cover at 11am (during Sentinel-2 acquisitions), and absence of precipitations during the day and the previous day. The maximum difference between two dates was 28 days, and the average difference was 20 days. Climatic conditions on each measurement day are shown in Fig. 9. The only day with precipitation was May 11, with heavy showers at 5pm.

### 4.3 Leaf sampling, *in situ* measurements and conditioning

In each of the four cardinal directions, two sun-exposed leaves were sampled from the top of each tree, using a telescopic branch cutter (4 m long). Considering the height of the operators plus the height of the branch cutter, the samples were taken as high as possible, on the outside of the crown, at a height of 6 m. In this study we assumed that eight leaves per tree were sufficient to represent the entire tree. More specifically, if the tree crown showed significant heterogeneity in leaf color, we sampled the leaves to account for this heterogeneity.

The eight leaves from each tree were weighed together immediately after sampling using an electronic scale (digital pocket model, Steinberg Systems, Berlin, Germany) (precision: 0.01 g) to measure the fresh weight (FW).

At the same time, the center of each leaf was measured on the adaxial face using the Dualex 4 Scientific. The eight values obtained were averaged to obtain a single value each for the contents of chlorophylls, anthocyanins and flavonols and the nitrogen balance index (Cab_DX, ANTH_DX, FLAV_DX and NBI_DX, respectively).

The eight leaves were placed in a cooling box at 8 °C until laboratory analysis. The time from harvest to cooler never exceeded 5 min. The temperature of the cooler was controlled, and ice was replenished to maintain this temperature throughout the day.

### 4.4 Laboratory leaf measurements

The leaves were freeze-dried in a freeze dryer (Beta 1–8 LSCplus, CHRIST, Osterode am Harz, Germany). After drying, the eight leaves were weighed together using a fixed electronic scale (LE324S-0CE, Sartorius, Goettingen, Germany) (precision: 0.1 mg) to measure the dry weight (DW). The leaf dry matter content (LDMC) and leaf water content (LWC) were then calculated as follows:

$$LDMC=\left(\frac{DW}{FW}\right)\cdot 100$$

LWC=100−LDC

Then, two protocols were used to calculate pigment contents in the laboratory. To measure the contents of chlorophyll a, b, *a* + *b* and carotene (C_a_, C_b_, C_ab_ and Car, respectively), the dry leaves were first crushed, an aliquot of *ca*. 2 mg of crushed material was taken from each sample, and its mass was measured and diluted with 1 mL of 80 % acetone. Pigment extraction was carried out by incubating leaf powder with 1 mL of acetone 80 % for 1 hour at 4 °C under agitation (vortex).

The samples were then centrifuged for 10 min at 11 000 *g*, and the supernatant was collected. The absorbance of each sample (A) was then measured using a spectrophotometer (FLX Genius XM, SAFAS, Monaco) at 470, 646 and 663 nm. C_a_, C_b_ and Car contents (µg/mL) were calculated as follows [1]:

$$C_{a}=12.21\cdot A_{663}-2.81\cdot A_{646}$$

$$C_{b}=20.13\cdot A_{646}-5.03\cdot A_{663}$$

$$Car=\frac{1000\cdot A_{470}-3.27\cdot \text{Ca}-104\cdot \text{Cb}}{229}$$

The values obtained were then related to the volume of extraction (1 mL) and to the exact mass of aliquot used in order to express the chlorophyll and carotenoid concentrations as µg/mgDW.

Anthocyanin contents were determined using a 15 mg of leaf. The extraction protocol [2] comprised the following steps:

• Incubation of the powder in 400 µl of methanol for 15 min under agitation (vortex).
• Addition of 200 µl of chloroform and vortexing for 5 min at 4 °C.
• Addition of 400 µl of cold pure water and vortexing for 1 min at 4 °C.
• Centrifugation for 15 min at 14 000 *g* to separate the organic phase containing the chlorophylls from the upper water/methanol phase containing the anthocyanins.
• Taking of 700 µl of the aqueous phase and addition of 175 µl of 0.3 M hydrochloric acid. Anthocyanins were then quantified using a spectrophotometer (FLX Genius XM, SAFAS) by measuring the extract absorbance at 532 nm. Data were expressed as A_532_ /ml/mgDW.

### 4.5 Plant area density measurement

PAD was measured by two operators using two LAI-2200 plant canopy analyzers, with one measuring the clear sky in open environment (*i.e.*, above the canopy) and the other measuring below the canopy at the same time (Fig. 5). Before beginning the measurements, the two analyzers were calibrated together using the procedure in the LI-COR user manual (https://www.catec.nl/uploads/pdf/LAI-2200C%20Manual_2347.pdf) to ensure that they provided the same reading when measuring the clear sky. All measurements were taken at sunrise or sunset to avoid direct sunlight as much as possible, and with a 90° view cap on the analyzers to restrict the field of view away from the tree trunks.

For morning measurements, operators started measurements when there was sufficient daylight and artificial illumination sources (street lamps) were switched off and measurements continued until the sun was high enough to provide direct illumination. For evening measurements, the reverse procedure was used. Special care was taken to ensure that no measurements were taken when a car was passing with its headlights on, to avoid bias in measurements. Measurements at sites 1 and 4 were always taken in the morning, while those at sites 2 and 3 were always taken in the evening.

Each tree was measured four times, with the field of view oriented successively in each cardinal direction. Operators were equipped with a compass to ensure proper cardinal alignment. Below-canopy and above-canopy measurements were made simultaneously, operators communicating by walkie-talkie to ensure the simultaneity of the measurements. The same operator took the below-canopy measurements for all sites and dates. The distance from the trunk and the sensor altitude were standardized so that measurements were always taken one meter from the trunk and one meter from the ground according to Wei et al. 2020 [7] and practical considerations. The one-meter distance between the trunk and the sensor allows the operator to lean against the trunk and take constant measurements. The sensor altitude of one meter is the most appropriate with respect to the urban environment which contains many obstacles below one meter (street furniture, cars parked near trees, bushes, etc.), and with respect to the operators’ ability to handle the sensor on a horizontal plane. This measurement procedure for a single tree, described in the LI-COR user manual, was followed by Wei et al. 2020 [7] and Adeline et al. 2021 [8].

Since the height of a tree canopy varies (unlike that of a crop canopy), we calculated light-path lengths, which correspond to distances traveled within the crown, for each of five angles (θi) before calculating PAD. Light-path lengths were calculated using two crown cross-sections per tree, one oriented west-east and one oriented north-south. The crown cross-section was calculated using a canopy-height model normalized with a digital terrain model with an altimetric accuracy of 20 cm, both of which being referenced as primary data in the specification table section, the vector layer of tree-crown extent and the point layer with the locations of the tree trunks. First, two canopy-height profiles were extracted using the canopy-height model based on two perpendicular transects (Fig. 11a), and each profile was then closed with a straight line to form a polygon (Fig. 11b). Second, because the profiles provided only the top of the canopy crown cross-section, they were completed to estimate the entire crown cross-section. A live crown ratio, which equaled the ratio of crown height to total tree height, was set by default at 0.66 and used to estimate the theoretical crown base height (TCBH). If the predicted base crown height of the profile was higher than the TCBH, the crown cross-section was estimated as an ellipsoid truncated at the TCBH (Fig. 11c). If it was lower than the TCBH, it was used as the crown base height (Fig. 11d). To avoid underestimating the crown base height, we set its minimum height at 2 m (at which the trees were cut to avoid disturbing pedestrians).

**Fig. 10:**
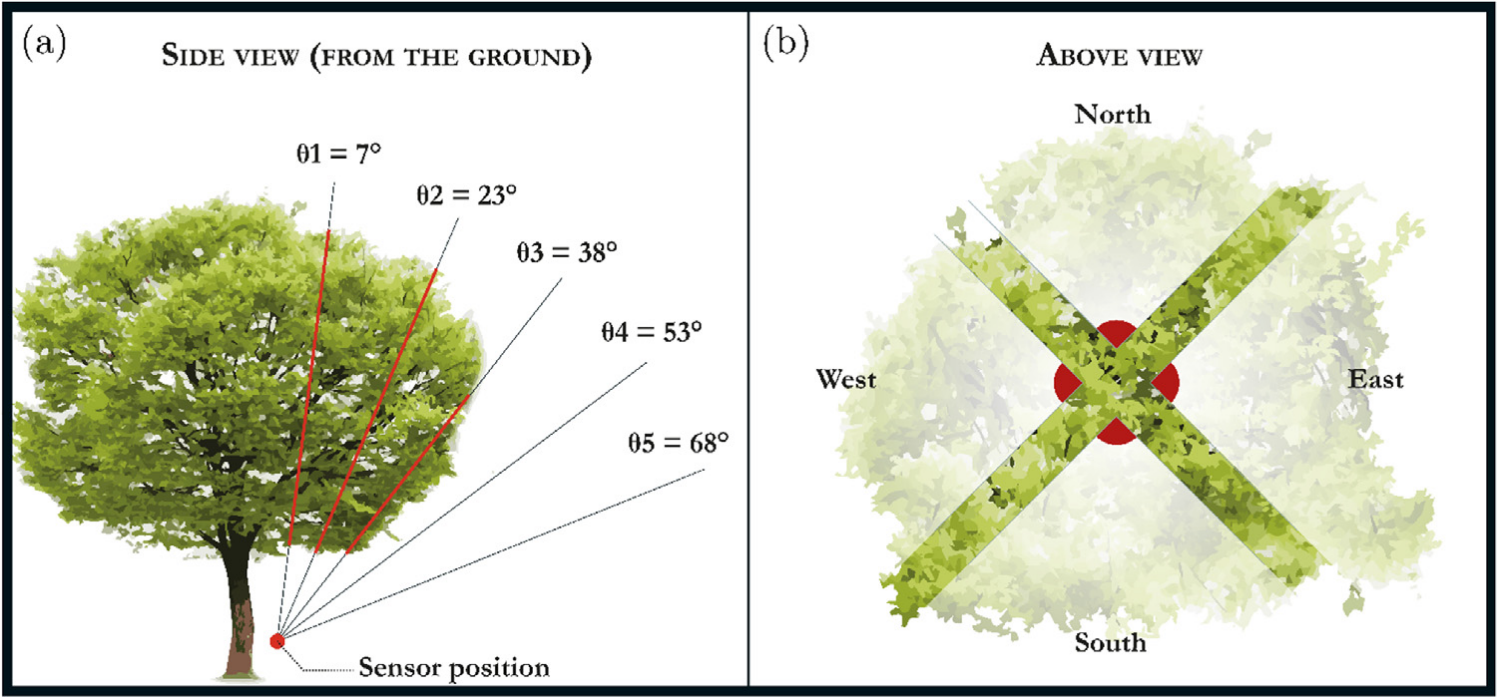
Graphical representation of the measurements taken to calculate plant area density using plant canopy analyzers. a- Side view from the ground (red lines indicate light paths that intersect the canopy); b- Above view.

The path lengths were calculated from the crown cross-sections at each angle θi (Fig. 10). When an angle did not intersect the canopy, it was excluded from calculations. For each pair of LAI-2200 measurements (above and below the canopy), the distances were integrated to calculate the PAD using the four methods included in the FV-2200 software.

**Fig. 11:**
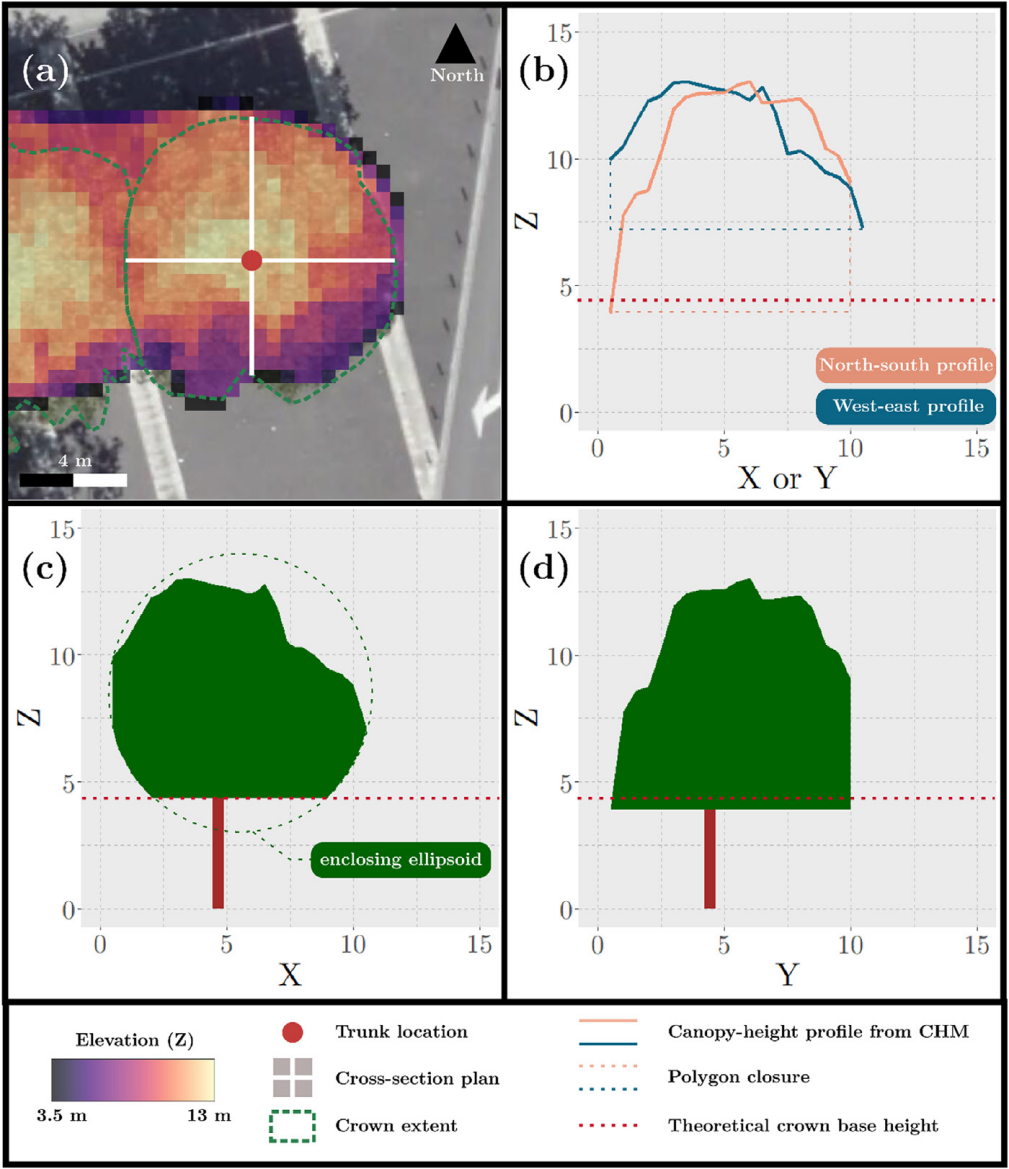
Graphical representation of how the crown cross-section was estimated. (a) Zenithal view of the two perpendicular transects; (b) The two crown height profiles from the canopy-height model (CHM), with the bottom line of each polygon corresponding to their respective profile's minimum height; The crown cross-section in the (c) west-east and (d) north-south directions.

## Limitations

Although special care was taken during the measurements with the LAI-2200, the crown modeling, which was based on simplifying assumptions, could have introduced bias, especially in the definition of the lower part of the tree crown: (i) the default live crown ratio was set to the average value of 0.66, whereas this ratio can be highly dependent on the tree species; (ii) the ellipsoidal shape chosen to represent the lower part of the crown is a simplified representation that corresponds to the majority of the trees studied. However, it is important to note that this shape may not accurately represent all trees. While these assumptions may introduce bias, they remain consistent for an individual tree across all dates. Consequently, the observed dynamics are independent of the potential bias associated with the lack of geometric precision. However, the crown geometry was defined at a single date in 2021 and did not account for branch growth throughout the growing season. Moreover, excluding angles that don't intersect the canopy may lead to biased results when a significant portion of angles are excluded for tree crowns with an asymmetric shape. Another limitation is that the dataset did not include soil moisture data, because in the vast majority of cases the soil surrounding the trees did not allow measurements to be taken using a moisture sensor such as TDR (time-domain reflectometry due to imperviousness or excessive compaction).

## Ethics Statement

Ethics statements do not apply to the dataset since it does not involve the use of human subjects, animal subjects or social media resources.

## CRediT Author Statement

**Théo Le Saint:** Visualization, methodology, investigation, writing - original draft; **Jean Nabucet**: Conceptualization, methodology, investigation, project administration, funding acquisition, supervision; **Cécile Sulmon**: Conceptualization, methodology, investigation, resources; **Julien Pellen**: Investigation; **Karine Adeline**: Conceptualization, investigation, supervision; **Laurence Hubert-Moy**: Supervision, writing - review & editing.

## Data Availability

zenodoA spatio-temporal dataset for ecophysiological monitoring of urban trees (Original data)
